# Exposure to dietary fatty acids oleic and palmitic acid alters structure and mechanotransduction of intestinal cells in vitro

**DOI:** 10.1007/s00204-023-03495-3

**Published:** 2023-04-29

**Authors:** Janice Bergen, Martina Karasova, Andrea Bileck, Marc Pignitter, Doris Marko, Christopher Gerner, Giorgia Del Favero

**Affiliations:** 1 Department of Food Chemistry and Toxicology, Faculty of Chemistry, University of Vienna, Währingerstr. 38-42, 1090 Vienna, Austria; 2 Core Facility Multimodal Imaging, Faculty of Chemistry, University of Vienna, Währingerstr. 38-42, 1090 Vienna, Austria; 3 Department of Analytical Chemistry, Faculty of Chemistry, University of Vienna, Währingerstr. 38-42, 1090 Vienna, Austria; 4 Joint Metabolome Facility, University of Vienna, Medical University of Vienna, Vienna, Austria; 5 Department of Physiological Chemistry, Faculty of Chemistry, University of Vienna, Josef-Holaubek-Platz 2, 1090 Vienna, Austria

**Keywords:** Intestinal toxicity, HCT116, HCEC-1CT, Palmitic acid, Oleic acid, Mechanosensory apparatus

## Abstract

Intestinal cells are continuously exposed to food constituents while adapting to peristaltic movement and fluid shear stress. Oleic acid (OA) and palmitic acid (PA) are among the most prevalent fatty acids with respect to dietary lipids. Despite the central importance of dietary lipids for a balanced diet, awareness about potential detrimental effects related to excessive consumption is increasing; this includes toxicity, metabolic deregulation, and, particularly for cancer cells, a benefit from the uptake of fatty acids related to promotion of metastasis. Expanding on this, we started elucidating the effects of OA and PA (25–500 µM) on non-transformed human intestinal epithelial cells (HCEC-1CT) in comparison to colon carcinoma cells (HCT116), with regard to the mechanosensory apparatus. Hence, intestinal cells’ motility is on the one side essential to ensure adaption to peristaltic movement and barrier function, but also to enable metastatic progression. Incubation with both OA and PA (≥ 25 µM) significantly decreased membrane fluidity of HCT116 cells, whereas the effect on HCEC-1CT was more limited. Application of rhodamine-labelled PA demonstrated that the fatty acid is incorporated into the plasma membrane of HCT116, which could not be observed in the non-tumorigenic cell line. Down-streaming into the intracellular compartment, a pronounced rearrangement of actin cytoskeleton was evident in both cell lines (OA and PA; 25 and 100 µM). This was accompanied by a variation of translocation efficiency of the mechanosensitive co-transcription factor YAP1, albeit with a stronger effect seen for PA and the cancer cells. Untargeted proteomic analysis confirmed that exposure to OA and PA could alter the response capacity of HCT116 cells to fluid shear stress. Taken together, OA and PA were able to functionally modulate the mechanosensory apparatus of intestinal cells, implying a novel role for dietary fatty acids in the regulation of intestinal pathophysiology.

**Supplementary Information:**

The online version contains supplementary material available at 10.1007/s00204-023-03495-3.

## Introduction

Palm oil, derived from the oil palm (*Elaeis guineensis)*, has been a topic of great controversy over the past decades. The market for palm oil accounts for approximately a third of oil markets globally, making it by far the leading vegetable oil but also the crop with the highest oil yield (Vijay et al. 2016). Following the expanding request, initiatives to promote economic fairness and environmental protective measures have been implemented such as the Roundtable on Sustainable Palm Oil (RSPO) (Choiruzzad et al. 2021). Indeed, uses of palm oil range broadly from biofuel over cosmetics to the food industry. Approximately 85% of palm oil is used for food products, such as margarine, cereal, sweets, or baked goods (Abubakar et al. 2021; Sundaraja et al. 2021). Palm oil is rich in saturated fat and consists of 44% palmitic acid (PA) and 39.2% oleic acid (OA) (Mancini et al. 2015). Regardless the source, it is clear that dietary exposure to OA and PA is quite considerable; however, many aspects of their molecular mechanism of action or toxic potential remain unknown. Particularly, very limited knowledge is available on possible beneficial or detrimental effects of dietary oleic and palmitic acids on the motility and biomechanical compliance of intestinal cells. As a matter of fact, the intestinal epithelium is continually exposed to physical forces such as the compression and stretch stemming from peristaltic movements and withstands shear stress caused by the flow of luminal contents. To ensure epithelial homeostasis, further mechanical tasks are performed by the epithelium itself, including cell proliferation and extrusion, preservation of barrier integrity, transformation by crypt fission and fusion, tissue folding, migration between tissue compartments, and the partitioning of cell types (Perez-Gonzalez et al. 2022). The conversion of mechanical forces into biochemical signals—mechanotransduction (Tarbell and Pahakis 2006)—relies on a complex sensory apparatus, which upon perturbation can lead to pathophysiological conditions (Ando and Yamamoto 2022; De Felice and Alaimo 2020). On the cellular level, several components contribute to the conversion of mechanical stimuli encompassing the cell membrane, the actin cytoskeleton, mechanosensitive ion channels such as Piezo1, and mechanosensitive transcription factors. Centrally, the cell membrane acts as interface with the extracellular environment and its composition and structure lead to varying degrees of rigidity or fluidity, thereby influencing the functioning of signalling induced by mechanotransduction (Anishkin et al. 2014; Douguet and Honore 2019). Importantly, it was previously demonstrated that membrane biophysical properties and mechanosensitive Piezo1 channels’ expression and function can be modulated by dietary lipids (Rebhahn et al. 2022; Romero et al. 2019). As an example, exposure of human embryonic kidney (HEK) and mouse neuro-2a (N2A) cells to a variety of fatty acids including PUFAs, such as arachidonic acid (AA, C20:4), eicosapentaenoic acid (EPA, C20:5), and docosahexaenoic acid (DHA, C22:6) as well as margaric acid (MA, C17:0) modified cells’ membrane fluidity and function of the mechanosensitive Piezo1 ion channels. In this context, MA caused the membranes to become more rigid and displayed a higher bending stiffness compared to those enriched with PUFAs. (Romero et al. 2019). Additionally, the application of PA was described to modulate the shear stress-induced expression of peroxisome proliferator‑activated receptors (PPARs) in endothelial cells (Wang et al. 2021). Even if some lines of evidence infer for a role of dietary lipids in the regulation of intestinal biomechanical compliance, data available are very limited. On these molecular premises, a role for OA and PA in the regulation of the mechanosensory apparatus of intestinal cells was postulated. Assuming a differential sensitivity for non-transformed and tumour cells to dietary lipids (Mika et al. 2020), we started to investigate the effects of OA and PA on HCEC-1CT and HCT116 cells. Analysis was benchmarked on crucial elements constituting cells’ mechanosensory apparatus, including the plasma membrane (Nicolson and Ferreira de Mattos 2021) and actin cytoskeleton (Gouget et al. 2016; Reddy et al. 2013; Romero et al. 2020; Wang et al. 2022), but also Piezo1 channels (Beech and Xiao 2018; Fang et al. 2021; Ranade et al. 2015) and mechanosensory transcription factor YAP1 (Mohri et al. 2017; Piccolo et al. 2014; Totaro et al. 2018). To complete, uptake efficiency and functional effects on the response to physical cues exerted by fluid shear stress were chosen to mechanistically underpin the morphometric changes triggered by OA and PA in intestinal cell models.

## Materials and methods

### Treatment compounds

Palmitic acid [Sigma-Aldrich P0500-10G; 25–500 µM], oleic acid [Sigma-Aldrich O1383-1G; 25–500 µM], and cerulenin, *Cephalosporium caerulens* [EMD Millipore Corp M.W.223.3; 5–50 µM] and palmitic acid lissamine rhodamine [Avanti 2260669-55-2; 25 µM]. The study concentrations chosen for PA and OA (25–500 µM) were selected based on data previously published. In vivo fatty acid concentrations can range from approximately 100 µM to > 1 mM (Huber and Kleinfeld 2017). In vitro similar concentrations, 0–400 µM (Li et al. 2019) and 200 µM (Llor et al. 2003), have been previously used in model systems to mimic fatty acid exposure. Compounds were dissolved in DMSO, and solvent controls were matched to represent the same solvent concentrations after diluting the stock concentrations accordingly. FCS was substituted with 1 mg FA free BSA/mL medium (i.e. no serum) to support the uptake of the fatty acids without potential interference provided by the serum (Zunszain et al. 2003; Alsabeeh et al. 2018).

### Cell culture

The human colon cancer cell line HCT116 was acquired from ATCC and served as an in vitro model for cancerous intestinal cells. HCT116 cultures were performed according to the specification of the supplier using McCoys 5A medium (Gibco REF 22330-021) supplemented with 10% (v/v) fetal calf serum (FCS) 1% (v/v) Penicillin/Streptomycin in TC-Flasks (Sarstedt). The immortalized human colonic epithelial cell line HCEC-1CT was kindly provided by Prof. Jerry W. Shay (UT Southwestern Medical Center, Dallas, TX, USA). HCEC-1CT cultures were performed according to previously described protocol (Rebhahn et al. 2022; Warth et al. 2016) using Dulbecco’s Modified Eagle Medium 92.8% (Gibco, REF 21063-029), Medium 199 (10x) 2%, Cosmic Calf Serum 2%, 1 M HEPES buffer solution 2%, Insulin–Transferrin–Selenium [100 µg/ml] 1.04%, Gentamicin Solution 0.12%, Epidermal Growth Factor [100 µg/ml] 0.02%, and Hydrocortisone [5 mg/ml] 0.02%. For cultivation and incubations, humidified incubators were used at 37 °C and 5% CO_2_. For assays, cells were grown to 80–90% confluency.

### Cell viability

Viable cells are determined by the cleavage of the tetrazolium salt WST-1 (4-[3-(4-Iodophenyl)-2-(4-nitro-phenyl)-2H-5-tetrazolio]-1,3-benzene sulfonate) to formazan through cellular enzymes (Peskin and Winterbourn 2000, 2017; Tan and Berridge 2000). Cells were seeded into a 96-well plate (Sarstaedt, 83.3924) and incubated with palmitic acid (25–500 µM), oleic acid (25–500 µM), or cerulenin (5–100 µM) for 24 h. Cells were washed with phenol red-free DMEM. Then, 100 µL of WST-1 solution (WST-1:phenol red-free DMEM, 1:20) was added to each well and incubated for 30 min at 37 °C, 5% CO_2_. Thereafter, the absorbance was measured via plate reader (CYTATON 5762 plate reader, Biotek Instruments, Winooski, VT, United States) at 450 nm against a background blank. The reference wavelength was set to 650 nm. Three independent cell preparations were prepared, and three technical replicates were measured for each experimental condition.

### Membrane fluidity assay

As previously described (Del Favero et al. 2018; Zhang et al. 2011), a membrane fluidity assay was carried out. Cells were seeded into a 96-well plate with clear bottom (Thermo Scientific, 165305) and incubated with palmitic acid (25–500 µM), oleic acid (25–500 µM), and cerulenin (5–100 µM) for 24 h. Thereafter, cells were loaded with 1-pyrendecanoic acid (10 µM, PDA, Thermo Fisher Scientific, Waltham, MA, USA) for 1 h. Fluorescence was measured with a Cytation3 Imaging Multi-Mode Reader (BioTek, Winooski, VT, USA). After excitation at 344 nm, emission signals for PDA monomers (375 nm; *I*_*m*_) and excimers (470 nm; *I*_*e*_) were determined. Data are expressed as intensity ratios [excimer/monomer] and the graphs were obtained after performance of at least *n* = 3 biological experiments for each cell line and measurements performed in *n* = 3 technical replicates.

### Immunofluorescence

For the immunofluorescence studies, following antibodies were used: Piezo1 Polyclonal Antibody (Invitrogen, PA5-106296 dil 1:400), Rabbit monoclonal antibody to YAP1 (Abcam, Ab 52771 dil 1:700), and Alexa Fluor^™^ 568 donkey anti-rabbit IgG (H + L) (Invitrogen, A10042 dil 1:1000). Actin was counterstained with Oregon Green^®^488 phalloidin (Invitrogen, 07466 dil 1:500). To carry out the staining of the Piezo1 channels and YAP1 co-transcription factor, a previously implemented protocol by Del Favero et al. 2020 was adapted accordingly (Del Favero et al. 2020) and µ-Slide 8 well ibiTreat (ibidi 80,826) slides for both cell lines were prepared with cells reaching a confluence of 80% before use. Palmitic acid and oleic acid were applied at concentrations of 25 and 100 µM and cerulenin at 10 µM. Concentrations were chosen on the basis of the outcome of the cytotoxicity and membrane fluidity experiments. Following an incubation time of 24 h at 37 °C (CO_2_ 5%), the cells were fixed with formaldehyde (3.7% in PBS). For membrane Piezo1 staining blocking, 2% donkey serum (Sigma-Aldrich) in PBS-A was applied (1 h, room temperature, RT). The blocking mixture was removed and Piezo1 antibodies were applied. The antibodies were incubated at 4 °C overnight. On the following day, a series of washing steps were carried out with PBS-A only. Secondary donkey–anti-rabbit antibody was added for 1.5 h in the dark. Another series of washing steps were carried out. To enable cytoskeletal staining, cells were permeabilized for a short time with 0.2% TritonX-100 in PBS-A 200 µL (10 min at RT). This was followed by a second blocking step and addition of phalloidin (1.5 h at RT). Unbound reagents were removed washing with 0.05% TritonX-100 in PBS-A and with PBS-A only. Post-fixation was carried out using 3.5% formaldehyde in PBS-A. Hereafter, each well was washed with 250 µL PBS-A and PBS-glycine was added to quench the remaining formaldehyde for 5 min. Finally, mounting media with DAPI to counterstain cell nuclei was applied (Abcam ab104139). To preserve the staining until the imaging the slides were sealed and stored at 4 °C. For the staining of YAP1 protocol was slightly adapted and permeabilization was carried out as first step as previously described for other transcription factors (Groestlinger et al. 2022). Images for Piezo1 and YAP1, actin, and nuclei were acquired with LSM Zeiss 710 equipped with ELYRA PS.1 system with a Plan Apochromat 63X/1.4 oil objective (zoom 1.5). At least 3 images were taken from each well and single cell analysis was performed for at least 5 cells per well. Images were analysed with ZEN 2012 SP3 and FIJI. At least three independent cell preparations were analysed, and three technical replicates were measured for each cell line for both Piezo1 ion channels and YAP1 transcription factor independently. Cells were chosen from 9 optical fields, resulting in at least 250 quantified cells/ROIs (regions of interest). For the treatment of both cell lines with PA (25 µM, 100 µM) with and without serum and under shear stress conditions (3 h, 24 h, 2.8 dyn/cm^2^), the staining included only phalloidin as above. Imaging was carried out using the Lionheart FX automated microscope (BioTek Instruments Inc., Winooski, VT, USA) and six different optical fields were used for analysis for each condition resulting in the quantification of *n* = 18 optical fields from three independent cell preparations.

### Shear stress stimulation

To create shear stress *in vitro*, the MK3 control orbital shaker (IKA, Staufen, Germany) placed inside an incubator (37 °C, 5% CO_2_) was used. To this aim, cells were seeded in 6- or 12-well plates and were exposed for 3 h and 24 h orbital shaking corresponding to approximately 2.8 dyn/cm^2^ shear stress following previously described protocols (Bileck et al. 2021; Masiello et al. 2018; Warboys et al. 2019). This value was chosen on the basis of the stress response capacity of the two cell lines, as well as on the basis of reference values from literature describing the translocation of mechanosensitive transcription factors (Del Favero et al. 2018).

### Proteomics

#### Sample preparation

HCT116 cells were seeded in 6-well plates (Sarstedt) and after reaching 80–90% confluency were treated with OA or PA (100 µM). The treated cells and controls with no serum and 10% serum were exposed to static or shear stress conditions for 24 h. Protein concentration of cell lysates was determined using a BCA assay. For enzymatic protein digestion, 20 µg of protein was used and an adapted version of the EasyPhos workflow was applied (Humphrey et al. 2018). Briefly, protein reduction and alkylation were performed in one step using 100 mM TCEP and 400 mM 2-CAM, respectively. Subsequent enzymatic digestion was achieved by adding a Trypsin/Lys-C mixture (1:100 Enzyme-to-Substrate ratio) at 37 °C for 18 h. Afterwards, peptide solution was first dried to approximately 20 µL, mixed with loading buffer containing 1% TFA in isopropanol, and loaded on SDB-RPS StageTips to desalt the peptide sample. After washing twice, peptides were eluted with 60% ACN and 0.005% ammonium hydroxide solution, and dried and stored at – 20 °C until LC–MS analyses.

#### LC–MS/MS analysis

LC–MS/MS analysis was performed as described previously (Kovarik et al. 2022; Seiser et al. 2021). In short, dried peptide samples were reconstituted by adding 5 µL of 30% formic acid (FA) containing 4 synthetic standard peptides and subsequent diluted with 40 µL of loading solvent (97.9% H2O, 2% ACN, 0.05% trifluoroacetic acid). Thereof, 2 µL were injected into the Dionex Ultimate 3000 nano-high-performance liquid chromatography (HPLC) system (Thermo Fisher Scientific). Peptides were pre-concentrated on a pre-column (2 cm × 75 µm C18 Pepmap100; Thermo Fisher Scientific) with a flow rate of 10 µL/min using mobile phase A (99.9% H2O, 0.1% FA). Chromatographic separation was achieved on an analytical column [25 cm × 75 µm 25 cm Aurora Series emitter column (Ionopticks)] by applying a flow rate of 300 nL/min and using a gradient of 8% to 40% mobile phase B (79.9% ACN, 20% H2O, 0.1% FA) over 90 min, resulting in a total LC run time of 135 min including washing and equilibration steps.

Mass spectrometric analyses were performed using the timsTOF Pro mass spectrometer (Bruker) equipped with a captive spray ion source run at 1650 V. The timsTOF Pro was operated in the Parallel Accumulation-Serial Fragmentation (PASEF) mode by applying a moderate MS data reduction. The scan range (m/z) was set to 100–1700 and the 1/k0 scan range to 0.60–1.60 V.s/cm2 resulting in a ramp time of 100 ms to achieve trapped ion mobility separation. All experiments were performed with ten PASEF MS/MS scans per cycle leading to a total cycle time of 1.16 s. All samples were analysed as technical duplicates.

#### LC–MS/MS data analysis and evaluation

LC–MS/MS data analysis was performed using the publicly available software package MaxQuant 1.6.17.0 running the Andromeda search engine (Cox and Mann 2008). Raw data were searched against the SwissProt database “homo sapiens” (version 141219 with 20,380 entries) to achieve protein identification and subsequent label-free quantification (LFQ). Search parameter included an allowed peptide tolerance of 20 ppm, a maximum of two missed cleavages, carbamidomethylation on cysteins as fixed modification as well as methionine oxidation and N-terminal protein acetylation as variable modification. A minimum of two unique peptide per protein was set as search criterium for positive identifications. Furthermore, the “match between runs” option was applied, using a 0.7 min match time window and a match ion mobility window of 0.05 as well as a 20 min alignment time window and an alignment ion mobility of 1. An FDR ≤ 0.01 was set for all peptide and protein identification.

LC–MS/MS data evaluation as well as statistical analysis was accomplished using the Perseus software (version 1.6.14.0) (Cox and Mann 2012). All identified proteins were first filtered for reversed sequences as well as common contaminants and annotated according to the different study groups. Prior to statistical analysis, LFQ intensity values were transformed [log2(*x*)] and the mean of technical duplicates calculated. Thereafter, proteins were additionally filtered for their number of independent identifications (a minimum of 70% identifications in at least one group). Afterwards, missing values were replaced from a normal distribution (width: 0.3; down shift: 1.8). Two-sided t tests as well as statistics for volcano plots were performed applying an FDR of 0.05 and a S0 of 0.1, whereby S0 controls the relative importance of t-test p-value and difference between the means.

### Uptake of rhodamine-labelled palmitic acid

Experiments with rhodamine-labelled PA were performed using the same cell preparation protocol as for microscopy experiments. Treatment with rhodamine-labelled PA (25 µM) as well as controls were performed in presence of 0% serum or 10% serum. The rhodamine-PA solution was gently vortexed before application. The treatment was incubated for 24 h (37 °C, 5% CO_2_). After incubation the cells were washed and Live Cell Imaging Solution (LCI; Invitrogen™ A14291DJ) was added to each well before images were captured with LSM Zeiss 710 equipped with ELYRA PS.1 system with a C- Apochromat 63X/1.2 water objective. At least 3 independent cell preparations were prepared, and 3 technical replicates were imaged for each cell line resulting in a minimum of 9 optical fields for every experimental condition, resulting in at least 75 quantified cells/ROIs.

### Statistical evaluation

Statistical evaluation and creation of all graphs was performed using OriginPro, Version 2021. OriginLab Corporation, Northampton, MA, USA. One-way ANOVA with Fischer LSD for multiple comparison was performed for all data. Differences were considered significant applying a threshold value (p) of 0.05. A minimum of three independent cell preparations (biological replicates) were used for each experimental workflow.

## Results

### Membrane fluidity and cell viability

**Fig. 1:**
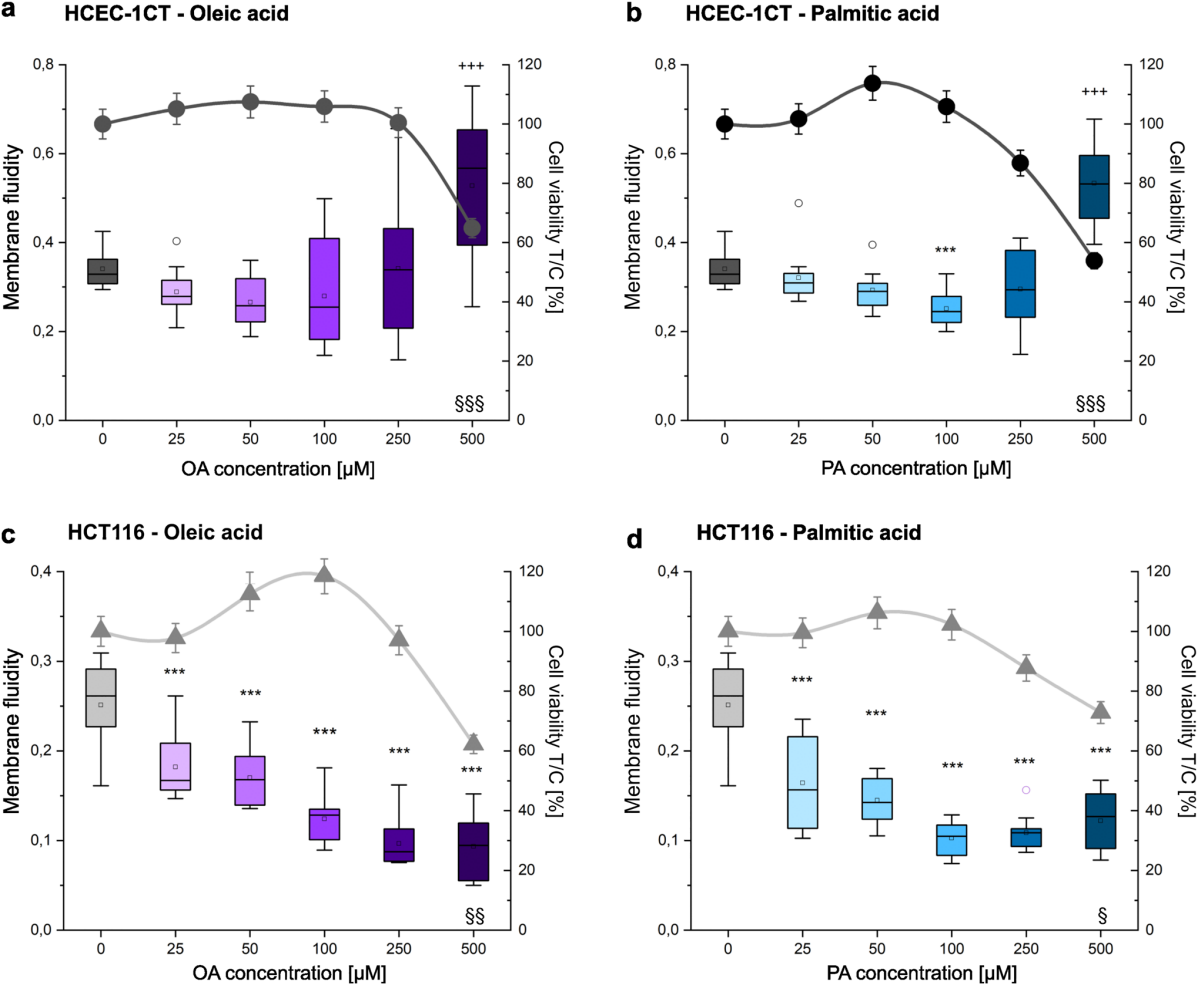
Effect of OA and PA on membrane fluidity and cell viability. The data of the membrane fluidity assay are expressed as intensity [excimer/monomer] as coloured boxplots (OA purple, PA blue, left *y*-axis); Cytotoxicity data are depicted as circles (HCEC-1CT) and triangles (HCT116) expressed as test/control [%] (right *y*-axis). Graphs **a** and **b** describe the HCEC-1CT cell line. Graphs **c** and **d** describe the cancer cell line HCT116. Error bars: standard error; boxes: 25/75 percentile with mean; °outliers. For cytotoxicity, data are derived from at least *n* = 3 biological replicates. For membrane fluidity, data are obtained from at least 9 wells per condition stemming from at least 3 independent cell preparations (biological replicates). Significant difference to control with ^***/^***p < 0.001 for membrane fluidity; §*p* < 0.05, §§*p* < 0.01, §§§*p* < 0.001 for cell viability (ANOVA test)

Appertaining to the “force from lipid” principle (Teng et al. 2015), it stood to determine whether OA and PA would induce alterations in the rigidity or fluidity of the plasma membrane, which would in turn affect the mechanical properties of the cells. When comparing controls of both cell lines, it could be observed that the HCT116 cell line is less fluid than the HCEC-1CT cell line (Fig. 1). In the HCEC-1CT cell line, the treatment with OA led to no significant change in membrane fluidity, with exception of 500 µM which falls into the cytotoxic range (Fig. 1a). Furthermore, in HCEC-1CT cells, only the treatment with 100 µM PA led to a significant (*p* < 0.001) decrease in membrane fluidity (Fig. 1b). In HCT116 exposure to both PA and OA (25–100 µM) triggered a significant (p < 0.001) concentration-dependent decrease of membrane fluidity (Fig. 1c and d). The increase of fluidity observable at higher concentrations developed in parallel to the cytotoxic effect, as visible in the WST-1 assay (Fig. 1, OA and PA 500 µM).

### Mechanosensitive Piezo1 ion channels

**Fig. 2:**
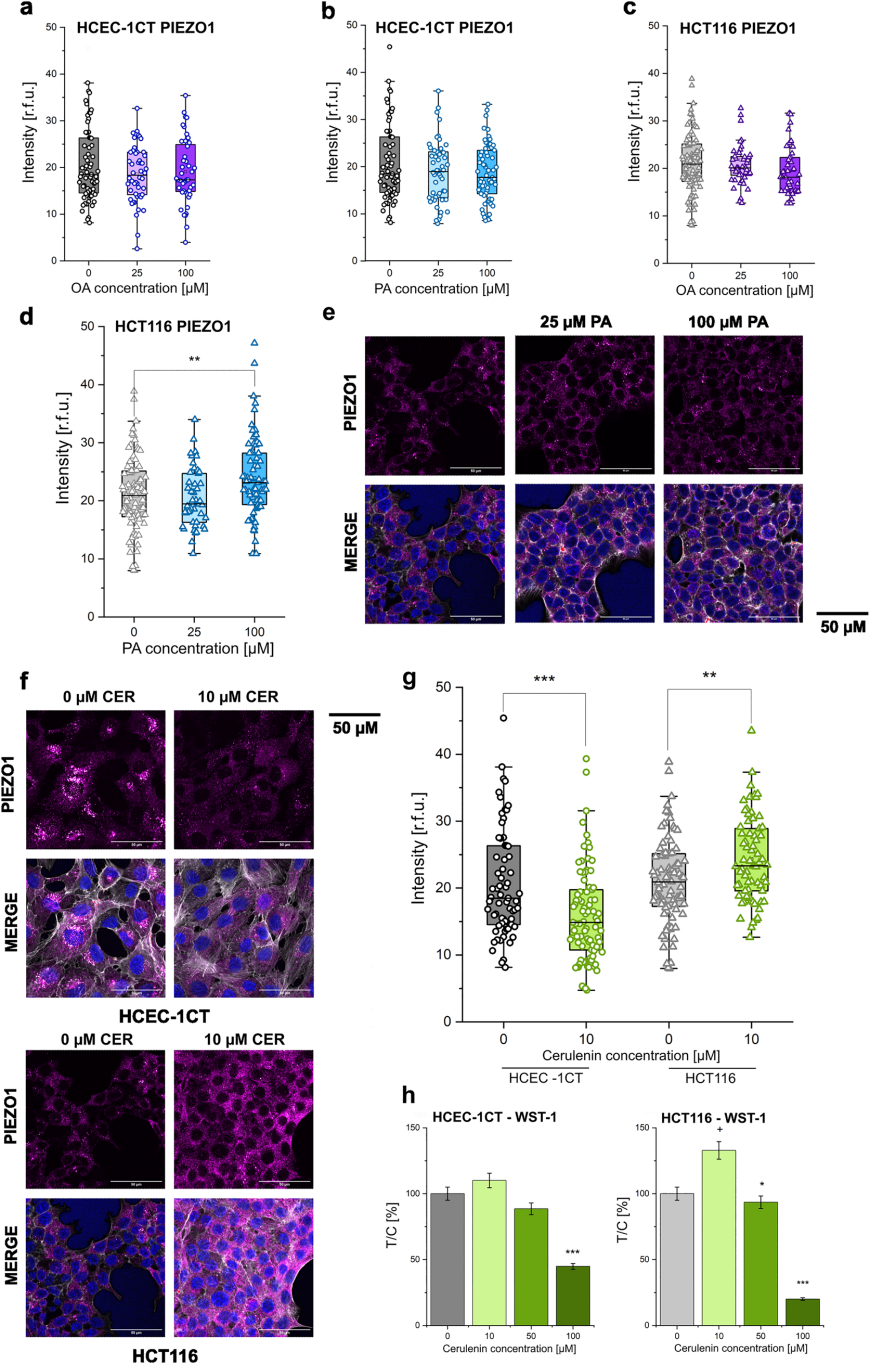
Effect of OA and PA on Piezo1. Treatments with PA and OA and resulting Piezo1 expression in HCEC-1CT cells (**a**, **b**, circles) and HCT116 cells (**c**, **d**, triangles). Images depicting the expression of Piezo1 in HCT116 cells after the treatment with PA (25 µM, 100 µM) compared to controls (**e**). Images of the expression of Piezo1 in both cells lines after treatment with 10 µM cerulenin (**f**) and corresponding Piezo1 intensity depicted as boxplots (**g** HCEC-1CT circles and HCT116 triangles). Cytotoxicity results for cerulenin in both cell lines (**h**). Merge: overlay between image acquisition depicting Piezo1, actin and nuclei. Data are expressed as intensity at 568 nm in relative fluorescent units [r.f.u.] and the graphs are obtained after performance of at least *n* = 3 biol. replicates in techn. n > 60 cells/ROIs; Significant difference to control with *^/^^+^*p* < 0.05, **p < 0.01, ***p < 0.001, *n.s.* no significance; Error bars: standard error; boxes: 25/75 percentile with mean (ANOVA test)

Considering the effects of OA and PA on the membrane fluidity, a possible effect on other components of the mechanosensory apparatus of the cells was investigated. As a first step, membrane mechanosensitive ion channels were considered. For HCEC-1CT incubation with OA and PA (25–100 µM) had no effect on the expression of the Piezo1 channels (Fig. 2a and b, Supplementary Fig. 1). There was likewise no significant difference to be observed for the treatment with OA in the cancer cells (Fig. 2c). However, the treatment with 100 µM PA in the HCT116 cell line led to an increase in the expression of Piezo1 ion channels (Fig. 2d and e, Supplementary Fig. 1). Since the response was relatively limited, we decided to verify in parallel the capacity of intestinal cells to adapt the expression levels of Piezo1 channels in response to modulation of lipid metabolism, to confirm a correlation between the two aspects and support the interpretation of the results obtained with PA. To this aim, cerulenin was used, namely an inhibitor of fatty acid synthase (FASN) which is a multienzyme complex whose primary function is to catalyse the production of PA from Malonyl-CoA and Acetyl-CoA in the presence of NADPH (Chirala et al. 2001; Smith 1994). Pharmacological modulation of lipid biosynthesis significantly modified the expression profile of Piezo1 in HCEC-1CT and also in HCT116 (Fig. 2f and g). In a non-cytotoxic concentration, cerulenin (10 µM, Fig. 2h) had opposing effects on the expression of Piezo1 and led to a significant (p < 0.01) increase of the ion channel detection in the HCT116 and a significant (*p* < 0.001) decrease in the HCEC-1CT cell line (Fig. 2f and g).

### Actin cytoskeleton and YAP1 transcription factor

**Fig. 3:**
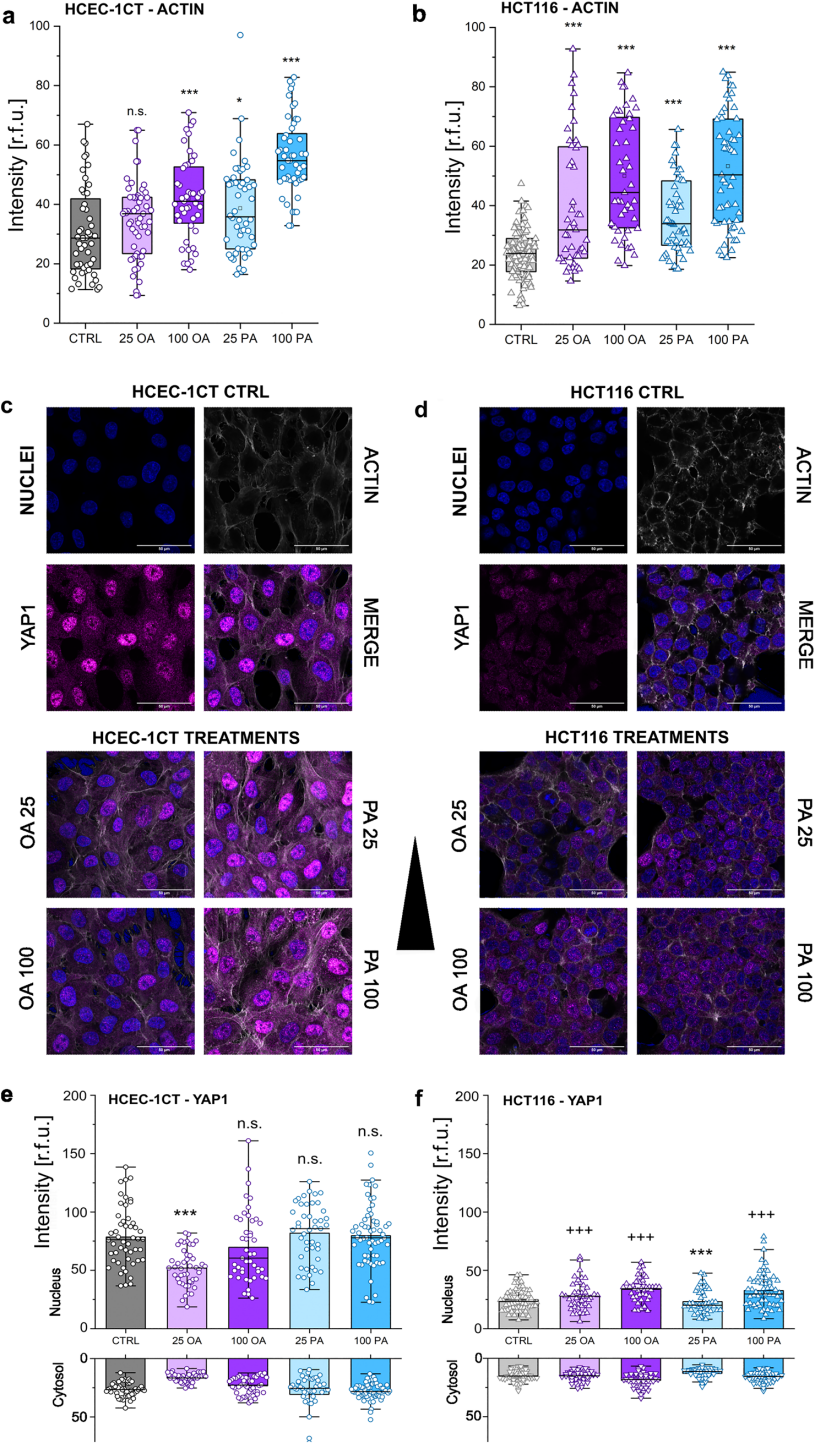
The effect of OA and PA on the actin cytoskeleton and YAP1 expression. Actin intensity is displayed for both cell lines after incubation with fatty acids (**a** and **b**, HCEC-1CT circles and HCT116 triangles). Representative images of YAP1 acquired after incubation with both OA and PA (25 µM and 100 µM) for HCEC-1CT (**c**) and HCT116 (**d**) cells. Distribution of YAP1 in the nucleus and cytosol is depicted for HCEC-1CT (**e**) and HCT116 (**f**) after treatment with OA and PA. Data are expressed as intensity of actin and YAP1 in relative fluorescent units [r.f.u.] and the graphs are obtained after performance of at least *n* = 3 biol. replicates in techn. *n* > 200 cells/ROIs; significant difference to control with *^/+^*p* < 0.05, **^/++^*p* < 0.01, ***^/+++^*p* < 0.001, *n.s.* no significance; error bars: standard error; boxes: 25/75 percentile with mean (ANOVA test)

In virtue of the continuous dynamic interplay between the membrane lipid rafts (MLR) and the underlying cytoskeleton, it was previously described that alteration of the cell membrane can “drag” the cytoskeletal elements when cells are exposed to mechanical stimuli (Del Favero et al. 2018). Hence, the effect of the incubation with PA and OA on the cytoskeleton was investigated. The treatment of HCEC-1CT and HCT116 cells with OA and PA was accompanied by adaptive changes of actin (Supplementary Fig. 2). In HCEC-1CT cells, an increase of actin signal was observed upon incubation with 100 µM OA and with PA ≥ 25 µM (Fig. 3a). In the cancer cell line, collectively a significant (*p* < 0.001) increase of actin signal could be identified for all treatments as compared to controls (Fig. 3b). Since significant changes in the cytoskeleton were demonstrable, we hypothesized that mechanosensitive transcription factors could potentially be translocated into the nucleus after incubation with OA and PA. Therefore, the transcription factor YAP1 (Supplementary Fig. 3) was selected for further experiments as, in addition of being mechanosensitive (Mohri et al. 2017), and its regulation was previously linked to the metabolism of lipids by way of the mevalonate/cholesterol pathway resulting in the promotion of cell proliferation (Wang et al. 2014). With exception of a significant decrease after incubating with 25 µM OA, (*p* < 0.001, Fig. 3e), there was no significant difference between the controls and the treatment conditions in the HCEC-1CT cell line with regard to expression of YAP1 (Fig. 3e). In the colon cancer cells treated with 100 µM of both OA and PA, a significant (*p* < 0.001) increase of YAP1 could be observed in the nuclear compartment. A concentration of 25 µM PA led to a significant (*p* < 0.001) decrease of YAP1 immunolocalization and a concentration of 25 µM OA conversely led to an increase (*p* < 0.05) of the transcription factor (Fig. 3f).

### Mechanosensitive proteome signature

**Fig. 4:**
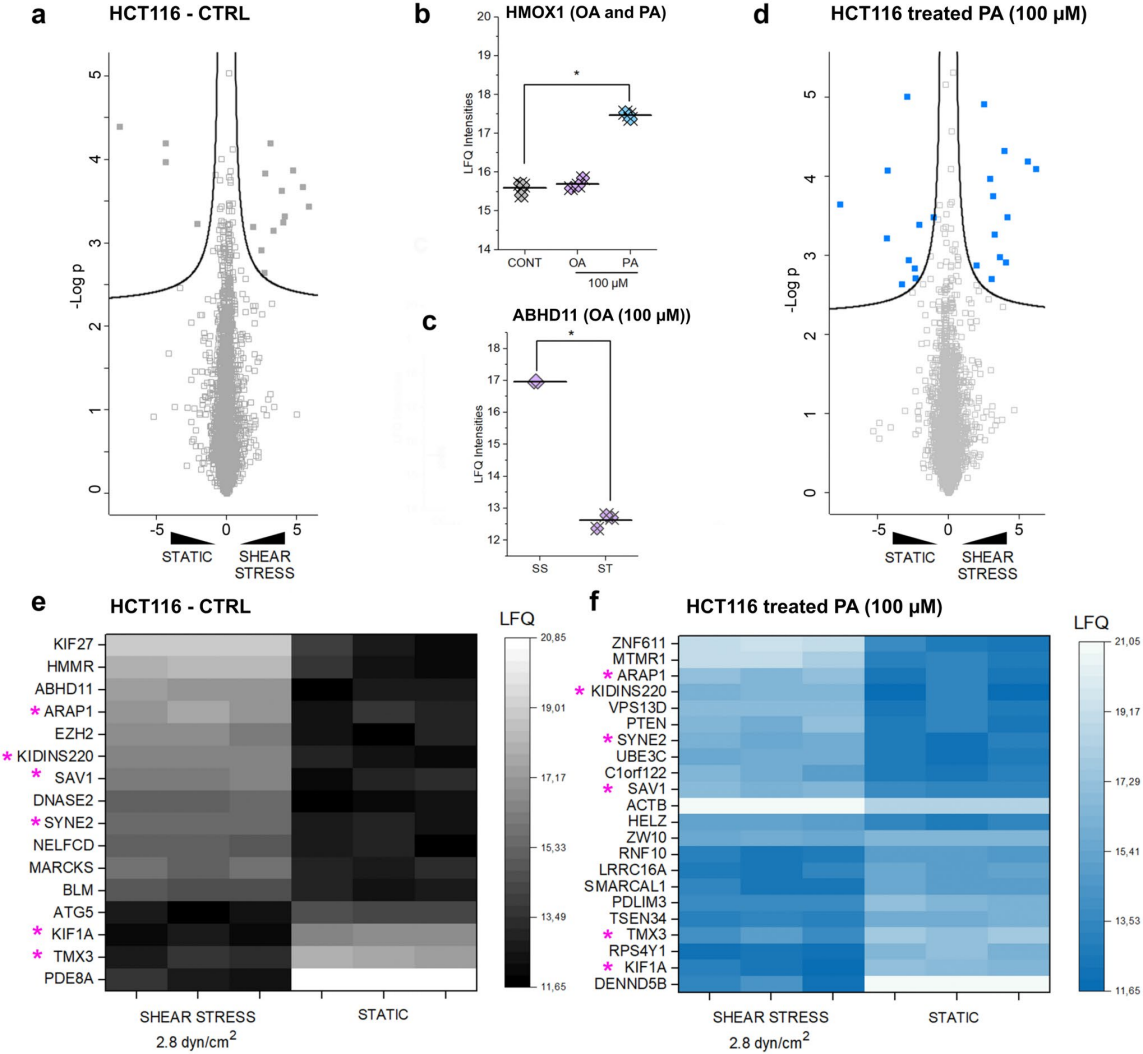
Effect of OA and PA on the proteome of HCT116 cells in static and shear stress conditions. Volcano plot depicting the upregulated proteins in static and shear stress conditions in the HCT116 controls (**a**). HMOX1 response profile in static incubation PA (blue), OA (violet), and controls (grey) (**b**). Regulation of ABHD11 after treatment with OA upon application of shear stress (SS) and in static conditions (ST, **c**). * depicts significant regulation among treatments. Volcano plot depicting the upregulated proteins in static and shear stress conditions in the HCT116 in presence of PA 100 µM (**d**). Heat maps depicting regulatory events in control cells (**e**) and PA-treated cells (**f**). Data depict LFQ intensities; *n* = 3. Marked by * (pink) commonly regulated proteins between the two signatures (color figure online)

To verify if the effects triggered by the regulation of mechanosensitive transcription factor YAP1 could also be related to the capacity of intestinal cells to respond to mechanical cues, a proof of principle proteomics experiment was performed in HCT116, comparing static culture to application of fluid shear stress. Indeed, based on the morphometric profiling, it remained open whether the measured effects triggered by OA and PA were limited to a structural adaption, or whether cells’ functional performance could be effectively modulated. On this basis, the data collected by the imaging experiments indicated a more pronounced response of the cancer cells to the treatments in comparison to HCEC-1CT, and therefore, HCT116 were prioritized for the measurement of the mechano-proteome. The proteome analysis resulted in the identification of 4745 proteins in the HCT116 samples. Principle Component Analysis (PCA) allowed to clearly distinguish the effects of the physical stimuli in the clustering of the treatment groups (Supplementary Fig. 4). With this experimental layout, it was possible to observe that in control conditions, 16 proteins were significantly regulated upon application of shear stress (2.8 dyn/cm2, Fig. 4a, Supplementary Table 1). Of these, 4 were downregulated, including the autophagy protein 5 ATG5, together with KIF1A, TMX3, and PDE8A (Fig. 4a and e, Supplementary Table 1). In addition, several proteins related to cell biomechanical compliance were upregulated, such as the Hyaluronan-mediated motility receptor (HMMR), Kinesin-like protein KIF27, the actin-binding proteins myristoylated alanine-rich C-kinase substrate (MARCKS) (Aderem 1992) and nesprin-2 (SYNE2) (Rajgor et al. 2012), and ARAP1 which contributes to the regulation of cell morphometric adaption and spread (Miura et al. 2002). Of note, SAV1 (Protein salvador homolog 1 (Mardin et al. 2010)), which is a component of the mechanosensitive Hippo pathway (Ma et al. 2019), was also significantly increased upon application of shear stress. Application of 100 µM OA or PA had a very limited impact on the proteome of HCT116 in static conditions, and hence, one protein was downregulated by incubation with OA (AKR7L) and only two were regulated by the presence of PA (WBSCR16 and HMOX1; Supplementary Table 2). As for the latter, the heme oxygenase 1 (HMOX1, Fig. 4b) is of particular mention, since it was previously described as part of the antioxidant response mechanisms triggered by PA (Shi et al. 2018). The presence of OA during the physical stimulation significantly reduced the number of regulated proteins in HCT116 in comparison to controls, and hence, only ABHD11 maintained its increased profile (Fig. 4c). On the other hand, incubation with PA increased the number of regulatory events detectable in the proteome and 22 proteins were found deregulated in this case (Fig. 4d and f, Supplementary Table 3). Of these, 6 retraced the signature already described for the control cells (ARAP1, KIDINS220, SAV1, SYNE2, KIF1A, and TMX3 Fig. 4e and f). Specific of the signature of PA were several proteins related to membrane structure and lipid interactions, endowed by the upregulation of the lipid phosphatases MTMR1 (Kim et al. 2002) and PTEN (Maehama and Dixon 1998) and of the intermembrane lipid transfer protein VPS13D (Fig. 4f). Additionally, cytoskeletal actin (ACTB, Fig. 4f) increased significantly. Pertaining the downregulated proteins (Fig. 4f, Supplementary Table 3), a significant difference was seen for ZW10 which is involved, among others, in the ER-to-Golgi membrane trafficking (Hirose et al. 2004), as well as for the F-actin-uncapping protein LRRC16A, the SWI/SNF-related matrix-associated actin-dependent regulator of chromatin subfamily A-like protein 1 (SMARCAL1) and for the cytoskeleton associated protein PDLIM3 (Lee et al. 2021). Significant downregulation was measured also for DENND5B, which is a member of the Rab GDP-GTP exchange factors (Yoshimura et al. 2010).

### Uptake response profile

**Fig. 5:**
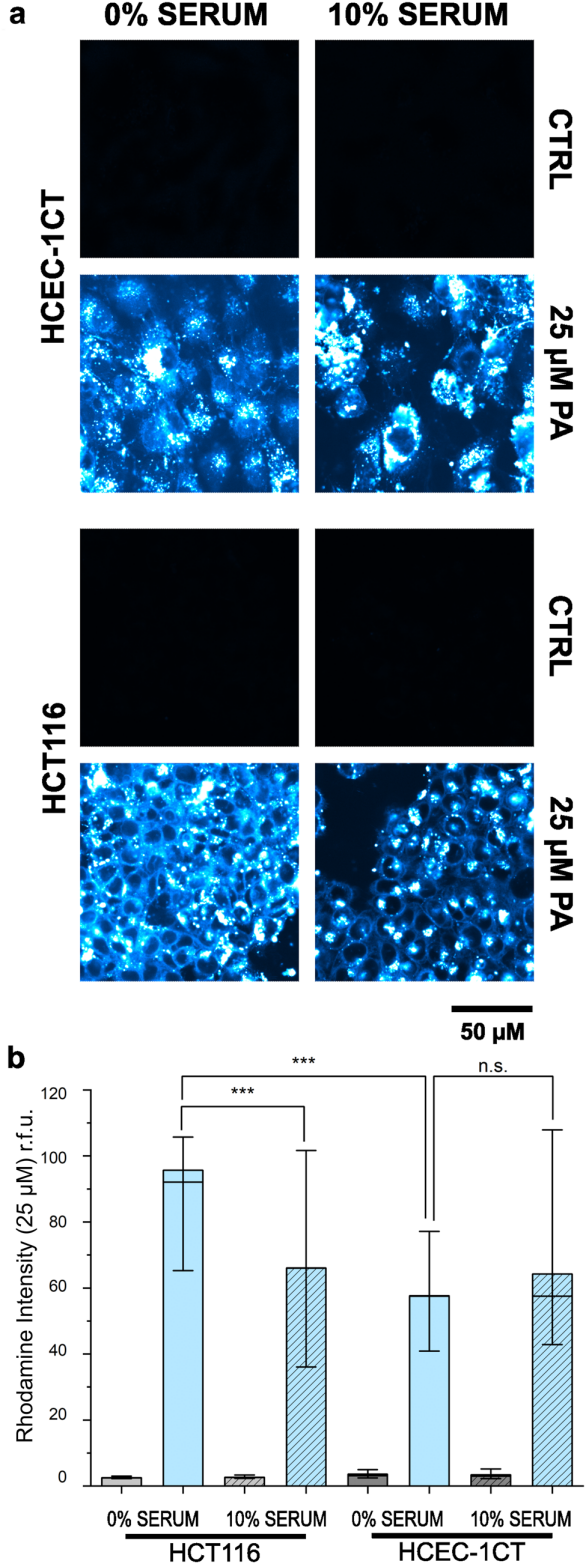
Uptake profile of PA into HCT116 cells compared to HCEC-1CT cells. **a** Representative images of the localization of rhodamine-labelled PA (25 µM, depicted in blue to white). Images of controls were captured to ensure the absence of autofluorescence (black panels, **a**). Rhodamine intensity in both cell lines in either 0% serum or 10% serum condition compared to controls (**b**) and expressed as relative fluorescent units [r.f.u.]. Data are derived from *n* = 90 cells/ROIs and expressed as mean values ± standard errors; significant difference to control with ***p < 0.001, *n.s* no significance (ANOVA test) (color figure online)

Since the increased response profile of the tumour cells HCT116 in comparison to non-transformed HCEC-1CT was quite consistent across the assays, we postulated that the cell lines could differ in their efficiency to incorporate the fatty acids. To verify this hypothesis, live-cell imaging experiments with fluorescently labelled PA (concentration 25 µM) were performed to observe the intracellular localization of the lipid. In line, it could be observed that the PA signal localised primarily on the cell borders, possibly incorporating into the cell membrane of HCT116 cells (Fig. 5a). This was accompanied by an intracellular accumulation of the rhodamine signal for the carcinoma cells (Fig. 5b). Incubation of HCEC-1CT cells returned a different uptake profile of PA in comparison to the cancer cells, with the PA localising almost exclusively in intracellular droplets, and no apparent accumulation at the cell membrane level. There was a significant (*p* < 0.001) difference between the uptake of PA in the two cell lines, with higher amounts of PA in the plasma membrane in the HCT116 cell line. Moreover, the uptake was more pronounced (*p* < 0.001) in the no serum condition as compared to the 10% serum condition in the cancer cell line, compatible with the hypothesis that during uptake of fatty acids, the serum lipids could compete with the labelled PA. As for the HCEC-1CT, the signal of the rhodamine-labelled PA was not influenced by the presence of the serum (Fig. 5b).

### Mechanosensitive cytoskeletal profiling

**Fig. 6:**
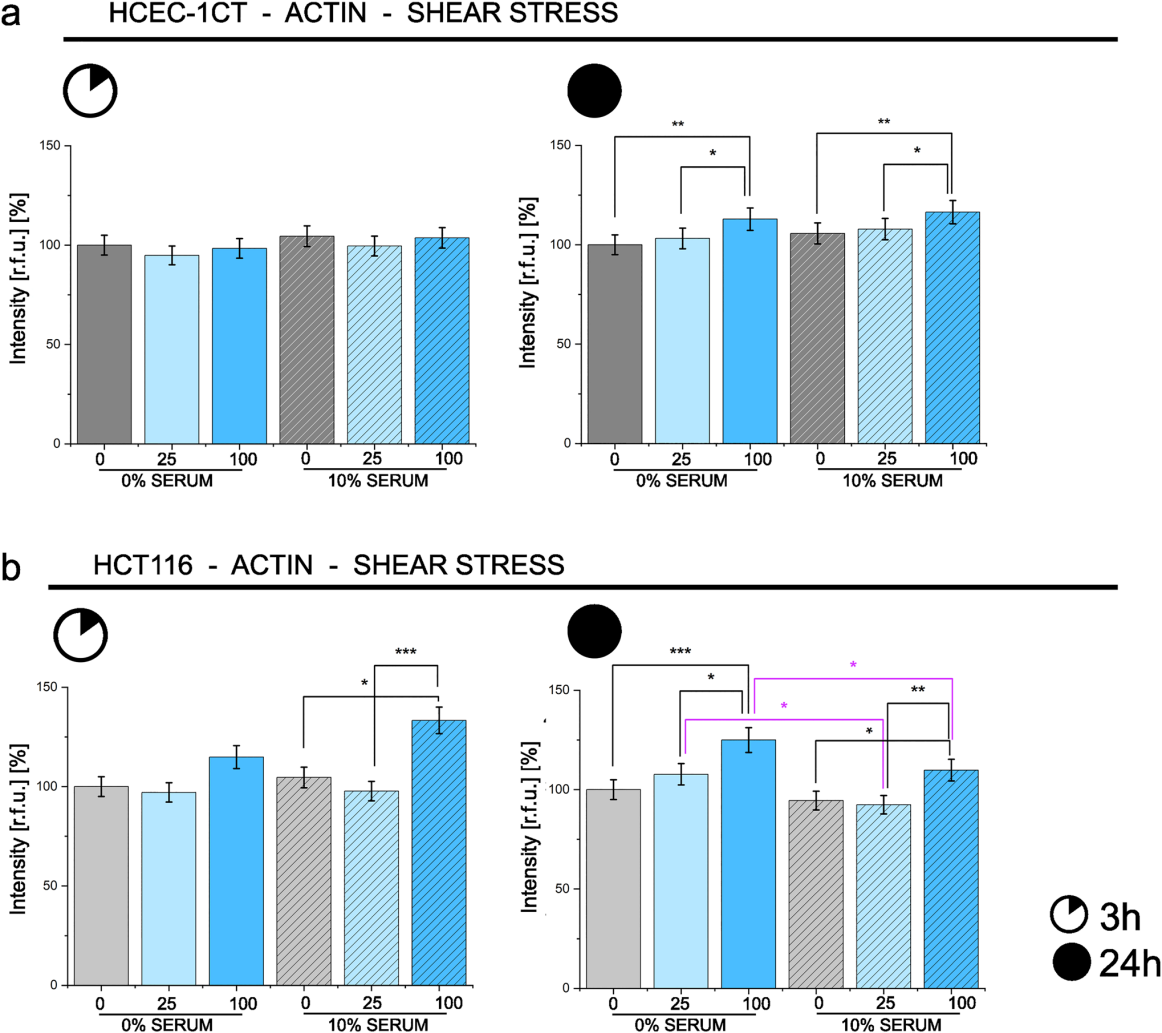
The effect of physical stimulation and PA on the cytoskeleton. Bar charts depict actin intensity [r.f.u.] [%] after shear stress (3 h and 24 h) in 0% serum (full bars) and 10% serum (diagonal pattern) in HCEC-1CT cells (**a**) and HCT116 cells (**b**). Treatment with 25 µM or 100 µM PA (blue) is compared to controls (dark grey and grey). Significant difference in comparison to controls or within treatment groups is depicted in black and the comparison between serum conditions (0% and 10% serum) in pink. *p < 0.05, **p < 0.01, ***p < 0.001 (ANOVA test). Data are derived from 3 independent cell preparations and the analysis of *n* = 18 optical fields for each condition (color figure online)

To verify if exposure to PA could be directly related to cellular capacity to adapt to physical stimuli, proof of principle experiments were performed applying PA (25 µM, 100 µM) with or without serum and challenging the intestinal cells with shear stress for 3 h or 24 h. Based on the proteome signature, actin cytosketon adaptive capacity was taken as reference for the activity profile. In line with the other datasets, HCEC-1CT showed moderate changes, with no response after 3 h stimulation and an actin signal increase after 24 h, which was not affected by the presence of serum (Fig. 6a and Supplementary Figs. 5–6). For the cancer cells, a more defined response profile could be observed (Fig. 6b and Supplementary Figs. 5–6). Cytoskeletal rearrangement was visible already after 3 h physical stimulation (100 µM PA, 10% serum, Fig. 6b) and consolidated after 24 h shear stress exposure (100 µM PA, 0% and 10% serum, Fig. 6b). In line with the results of the uptake experiments, for 24 h exposure, the presence of serum hampered the increase of the actin cytoskeletal signal fostered by the application of shear stress in presence of PA (24 h, Fig. 6b).

## Discussion

Worldwide colorectal cancer (CRC) is the second leading cause of cancer death (Xiang et al. 2022). In recent years, growing mortality rates have been observed and the global burden of CRC is estimated to increase by 60% to more than 2.2 million new incidences and 1.1 million deaths by 2030 (Arnold et al. 2017). This development can be related to known risk factors such as harmful foodborne exposures, lifestyle, stress, and aging populations (Kuipers et al. 2015). Furthermore, unfavourable dietary habits including high consumption of saturated fatty acids are linked not only to CRC, but also to numerous diseases such as type 2 diabetes (T2D), cardiovascular diseases (CVD), and metabolic syndrome. This contributes to an increase in morbidity and total mortality (Wang et al. 2016). The saturated fatty acid PA can be endogenously produced from other fatty acids, carbohydrates, or amino acids and does not necessarily need to be derived from dietary sources. In adipose tissue, PA also accounts for 20–30% of total fatty acids in triacylglycerols (TAG) and membrane phospholipids (PL) (Carta et al. 2015; 2017). High intake of PA is abrogated by PA endogenous synthesis via de novo synthesis ensuring that the tissue concentration does not significantly change (Song et al. 2017). If, however, de novo lipid biosynthesis is strongly activated through factors such as certain physiopathological conditions or nutritional constituents, an increase of PA in tissue and furthermore a disruption of the homeostatic control tissue concentration can be observed (Wilke et al. 2009). Typically, FFAs (free fatty acids) are bound to albumin in blood at concentrations ranging from 100 µM to > 1 mM. Due to the fact that most FFAs are bound, their total amounts do not necessarily directly mirror the physiologically active FFA concentrations. Unbound PA and OA can be found in the plasma at concentrations of approximately 135 µM and 370 µM, respectively (Huber and Kleinfeld 2017), thus in a concentration range which is represented in this study. Regardless of systemic uptake, the intestinal compartment is substantially exposed to dietary fatty acids; this raises the question if cells can adapt essential physiological functions, like those related to motility, to the presence of these molecules. Additionally, an increase in de novo fatty acid biogenesis is a metabolic attribute that equips tumours with proliferative and survival benefits, and therefore, FASN (fatty acid synthase) is overexpressed in many cancers, including colon cancer (Schroeder et al. 2021). Based on the pioneering findings of Romero and colleagues (Romero et al. 2019), describing the capacity of saturated margaric acid to tune the activity of mechanosensitive ion channels, we anticipated that PA could also potentially affect the mechanical compliance of the intestinal epithelium. Along this line, it is possible to hypothesize that OA might display a reduced or no effect due to the shorter chain length and only one double bond when compared to PUFAs. Consequently, looking for molecular pathways correlating the alteration of membrane fluidity as described in Fig. 1, a potential role for mechano-gated Piezo1 channels was investigated. In this case, only incubation with 100 µM PA returned a significant increase in the expression of Piezo1 (Fig. 2d). To confirm that, in the HCT116 model, fatty acid biosynthesis could be mechanistically related to the presence of Piezo1 channels, experiments were performed in presence of an inhibitor of fatty acid biosynthesis (Fig. 2f–h). In agreement with the postulated higher dependency of cancer cells from FASN, incubation with cerulenin was more toxic for HCT116 rather than for HCEC-1CT (Fig. 3h). This, on the one side, further underpinned previous data describing modified lipid metabolism and membrane structures between tumour and non-transformed intestinal cells (Del Favero et al. 2018; Li and Zhang 2016). Additionally, the inhibition of endogenous FASN returned a phenotype comparable to that elicited by presence of exogenous PA (Fig. 3d and g), sustaining the idea that lipid metabolism and expression of mechanosensitive ion channels could be mechanistically related. Supportive of the tight functional boundaries between membrane and cytoskeleton (Gefen and Weihs 2016), OA and PA elicited effects in both compartments. This is possible, because actin localises to the cortical or submembranal region where it supports the plasma membrane ensuring its integrity and stability (Aramaki et al. 2016). This interplay is vital for the regulation of many aspects in the functionality of eukaryotic cells with regard to their adaption to changing environments (Head et al. 2014). Importantly, in HCT116, both OA and PA displayed a rather coherent activity with reduction of membrane fluidity (Fig. 1c and d) accompanied by increase of actin cytoskeletal signal (Fig. 3b) and mobilization of YAP1 (Fig. 3f). However, only incubation with PA increased the proteins regulated upon application of physical stimuli (Fig. 4). This could be traced back to a major difference in the activity of the OA and PA with the selective enhancement of Piezo1 channels’ expression mediated by PA. Hence, it is known that Piezo1 levels of expression largely contributes to cell mechanical sensitivity (Millet et al. 2022). In agreement with a postulated major adaptive potential for the cancer cells, incubation with PA enhanced the response of HCT116 to physical stimulation (Figs. 4 and 6). 24 h shear stress combined to PA increased the actin signal detected by immunofluorescence (Fig. 6b) aligning to the results of the proteome where an upregulation of ACTB was accompanied by the increase of cytoskeletal ancillary proteins like the F-actin-uncapping protein LRRC16A, SMARCAL1, and PDLIM3 (Lee et al. 2021) (Fig. 4f, Supplementary Table 3). Besides, application of shear stress in presence of PA enhanced the expression of PTEN, but also of the E3 ubiquitin-protein ligase (UBE3C, Fig. 4f, Supplementary Table 3) in HCT116. This is particularly important; hence, it was previously described that PA could enhance the ubiquitin–proteasome mediated degradation of the PTEN, hampering in this way the role of the latter as tumour suppressor (Bai et al. 2021). Response of actin in HCEC-1CT was less influenced by the application of shear stress in presence of PA (Fig. 6a), which contributes to the general signature of this cell type as less responsive to fatty acid exposure. Cytoskeletal response of HCT116 and HCEC-1CT in presence of shear stress agrees also with the uptake profile of PA in the two cell lines: for HCT116, PA remained membrane-localised, whereas in HCEC-1CT accumulated in intracellular vesicles (Fig. 5). Intriguingly, PA uptake was reduced in HCT116 in the presence of foetal serum (10%, Fig. 5). This was most likely related to the high lipid content of the serum, which possibly hampers uptake efficiency. At the same time, this appeared an optimal tool to further investigate the relation between PA membrane localization and cell morphological adaption to shear stress. Indeed, in presence of foetal serum, actin increase stimulated by mechanical cues in presence of PA was reduced in HCT116 and not affected in HCEC-1CT (Fig. 6), retracing the outcome of the uptake experiments (Fig. 5). In line with a decreased responsiveness of the HCT116 model in presence of 10% serum (24 h incubation), untargeted proteome analysis performed in the same conditions revealed only 4 proteins significantly regulated after application of shear stress (upregulated VPS41 and SAV1; downregulated DENND5B and U2AF1L4, Supplementary Fig. 7 and Supplementary Table 4). Intriguingly, SAV1 was the protein which was upregulated in the response to mechanical cues in most experimental conditions (Fig. 4e and f, Supplementary Fig. 7 and Supplementary Tables 1,3,4). SAV1 regulates STK3/MST2 and STK4/MST1 in the Hippo signalling pathway which is composed of a kinase cascade ultimately leading to the phosphorylation and inactivation of YAP1 by LATS1/2. This inhibits the translocation of YAP1 into the nucleus to regulate genes pivotal for cell proliferation, cell migration, and cell death (Callus et al. 2006; Luo et al. 2009). Beside this mechanism and the aforementioned Hippo signalling pathway, the Wnt-pathway coordinates both ß-catenin and YAP/TAZ activity (Totaro et al. 2018). This pathway not only contributes to intestinal regeneration (Pinto et al. 2003), but is crucial for the early and late stages of colorectal cancer (Basu et al. 2016). In fact, the overactivation of Wnt signalling is one of the hallmarks of colorectal cancer, with aberrant Wnt activation in roughly 90% of intestinal tumours (Clevers 2006). Along this line, it appears plausible that HCT116 could adapt more extensively YAP1 expression to external stimuli, such as dietary OA and PA, as demonstrated experimentally (Fig. 3c–f).

## Conclusion

Taken together, the data generated in this study demonstrate that dietary fatty acids OA and PA influence the mechanosensory apparatus of intestinal cells. The overall effect was greater for PA and the colon cancer cell line HCT116 with regard to the modulation of the plasma membrane through the decrease of membrane fluidity and incorporation of PA into the membrane. This was related also to a change in the expression of the mechanosensitive Piezo1 ion channels (100 µM PA). Structural adaption proved functionally relevant, reflecting on the cell capacity to respond to shear stress. These data underline the difficulties to draw conclusive statements on the potential positive or detrimental effect of food constitutes, since it is clear that cells that are in a different physiopathological status are responding to the same molecule in a different fashion. Additionally, benchmarking our analysis on cell mechanotransduction we could describe molecular mechanisms of action that are measurable in a concentration range relevant for dietary exposure and way below the cytotoxic threshold and offer in this way a novel perspective for the interpretation of the bioactivity of dietary food constituents.

## Supplementary Information

Below is the link to the electronic supplementary material.

Supplementary file1 (PDF 1226 KB)

## Data Availability

Representative images obtained during microscopy experiments can be found in Supplementary Figures 1-2-3-5-6. Data used for the generation of Figure 4 are provided as Supplementary Tables 1-3. Further information about the datasets generated and/or analyzed during the study is available on request from the corresponding author.
