# Supplementary material for: Exposure to dietary fatty acids oleic and palmitic acid alters structure and mechanotransduction of intestinal cells in vitro
Source: Arch Toxicol. 2023 Apr 29;97(6):1659–75. doi: 10.1007/s00204-023-03495-3 (PMC10182945; doi:10.1007/s00204-023-03495-3)
Supplement: Supplementary file 1 — Supplementary file1 (PDF 1226 KB) [file 204_2023_3495_MOESM1_ESM.pdf]

Supplementary Materials

Exposure to dietary fatty acids oleic and palmitic acid alters structure and mechanotransduction of intestinal cells *in vitro*

Janice Bergen[1,2], Martina Karasova[1,2], Andrea Bileck[3,4], Marc Pignitter[5], Doris Marko[1], Christopher Gerner[3,4], Giorgia Del Favero[1,2*]

1 Department of Food Chemistry and Toxicology, Faculty of Chemistry, University of Vienna Währingerstr. 38-42, 1090 Vienna, Austria

2 Core Facility Multimodal Imaging, Faculty of Chemistry, University of Vienna Währingerstr. 38-42, 1090 Vienna, Austria

3 Department of Analytical Chemistry, Faculty of Chemistry, University of Vienna Währingerstr. 38-42, 1090 Vienna, Austria

4 Joint Metabolome Facility University of Vienna, Medical University of Vienna, Austria

5 Department of Physiological Chemistry, Faculty of Chemistry, University of Vienna, Josef-Holaubek-Platz 2, 1090 Vienna, Austria

* Correspondence to G.D.F. Giorgia.del.favero@univie.ac.at

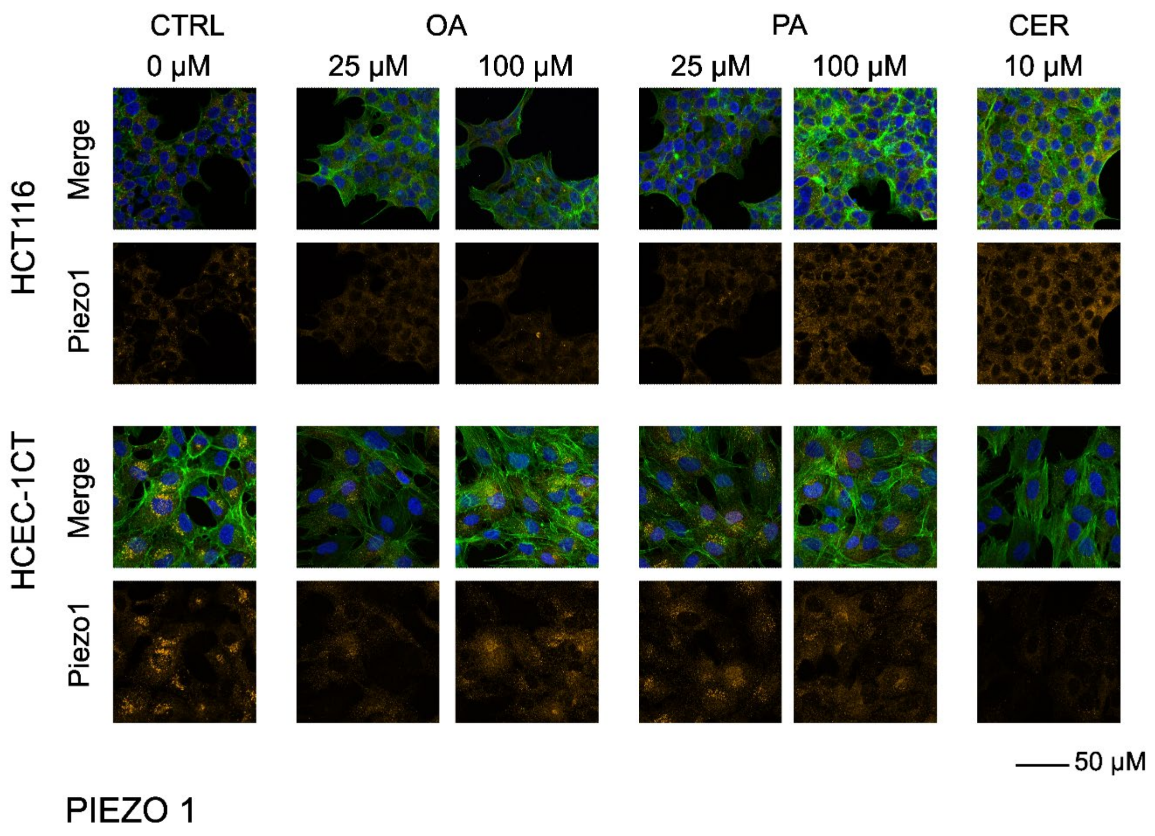

Supplementary Figure 1: Representative images of Piezo1 expression (orange) in HCT116 and HCEC-1CT cells. Actin cytoskeleton is depicted in green and cell nuclei in blue.

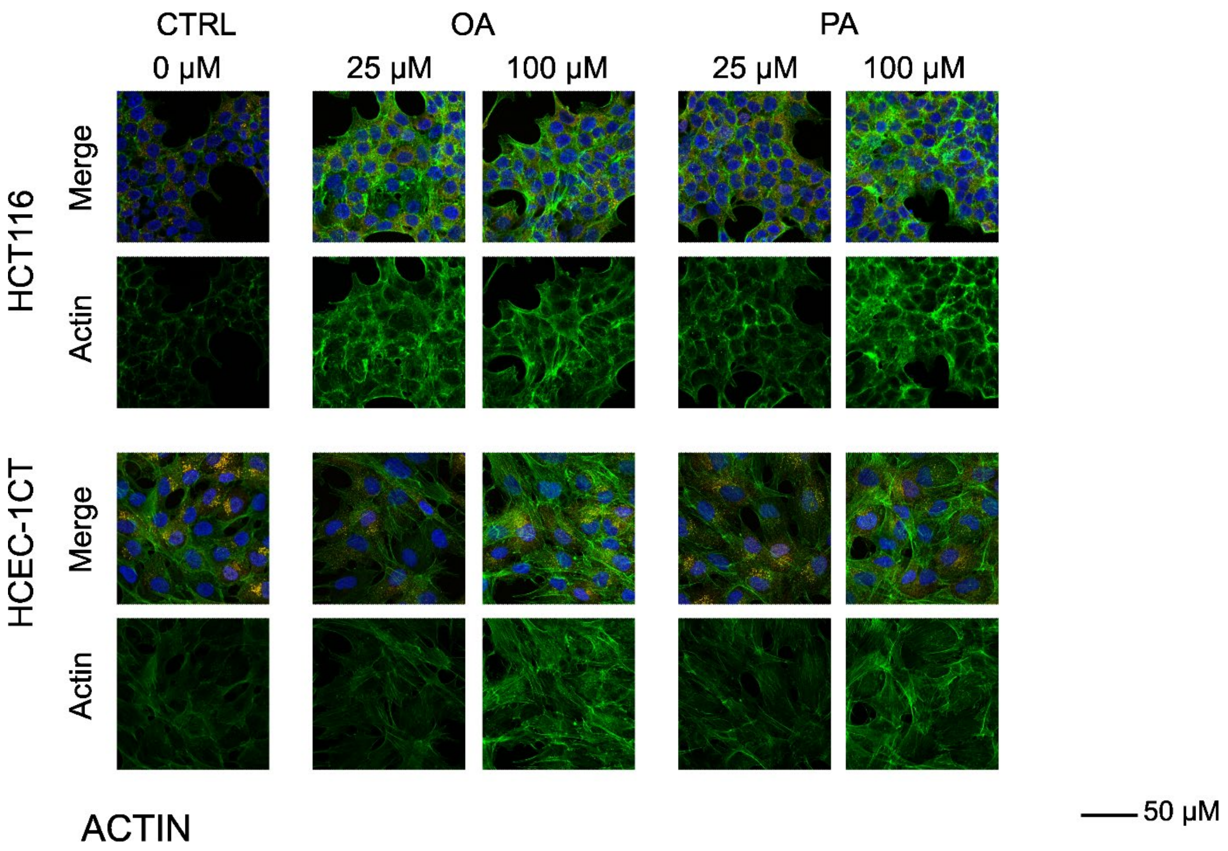

Supplementary Figure 2: Representative images of actin appearance in HCT116 and HCEC-1CT cells. Actin cytoskeleton is depicted in green and cell nuclei in blue.

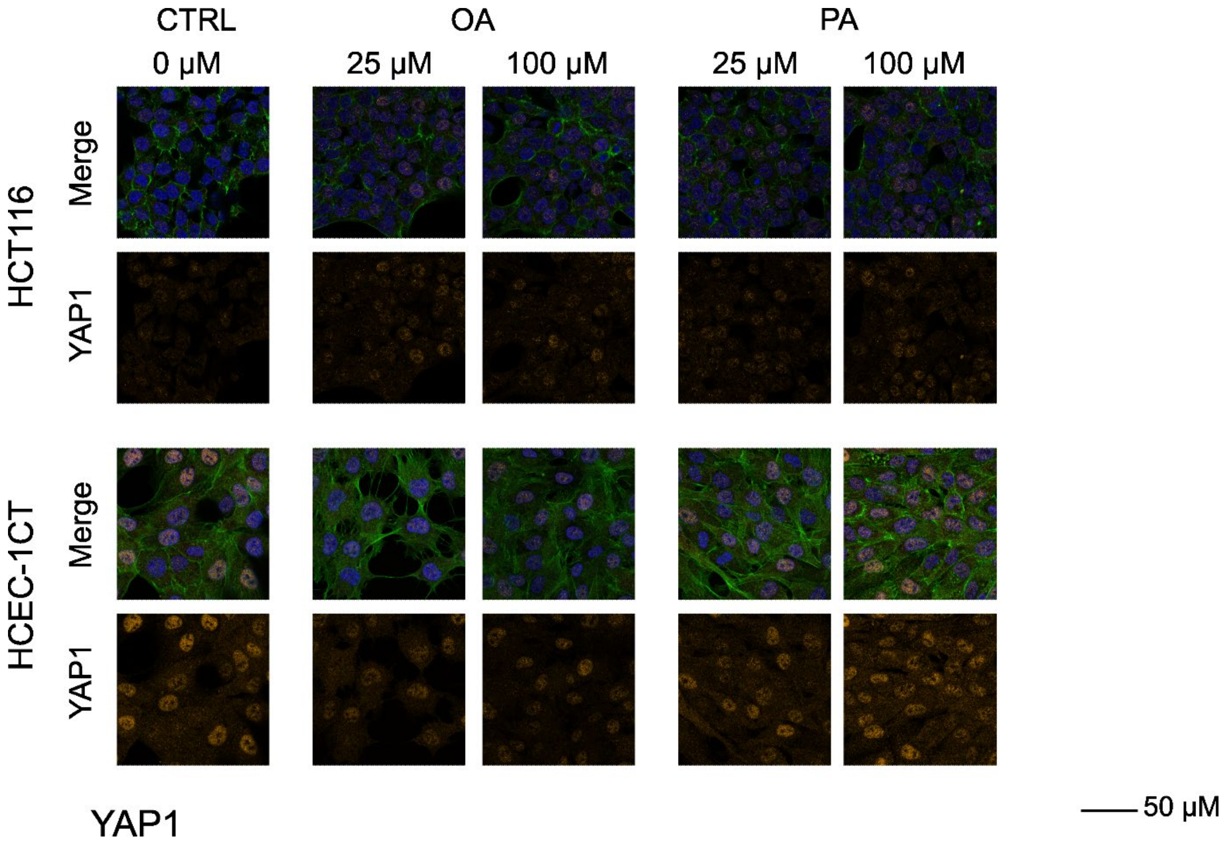

Supplementary Figure 3: Representative images of YAP1 expression (orange) in HCT116 and HCEC-1CT cells. Actin cytoskeleton is depicted in green and cell nuclei in blue.

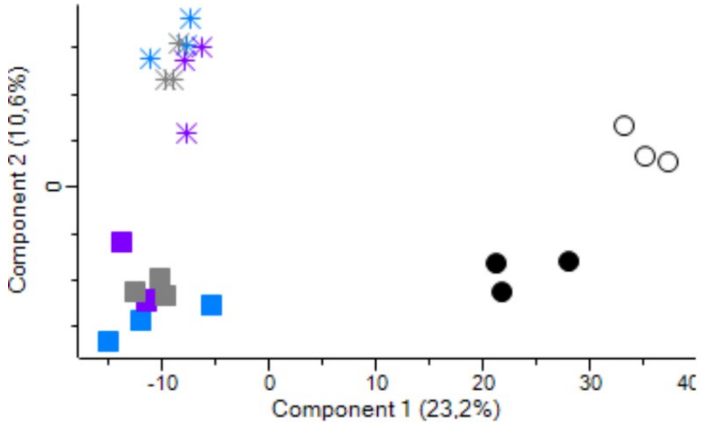

Supplementary Figure 4: PCA after treatment of serum deprived HCT116 cells with 100 µM OA (purple) and 100 µM PA (blue) and controls (grey) in static (squares) and shear stress (stars) conditions. Controls in presence of 10% serum in static (black circles) and shear stress (white circles) conditions.

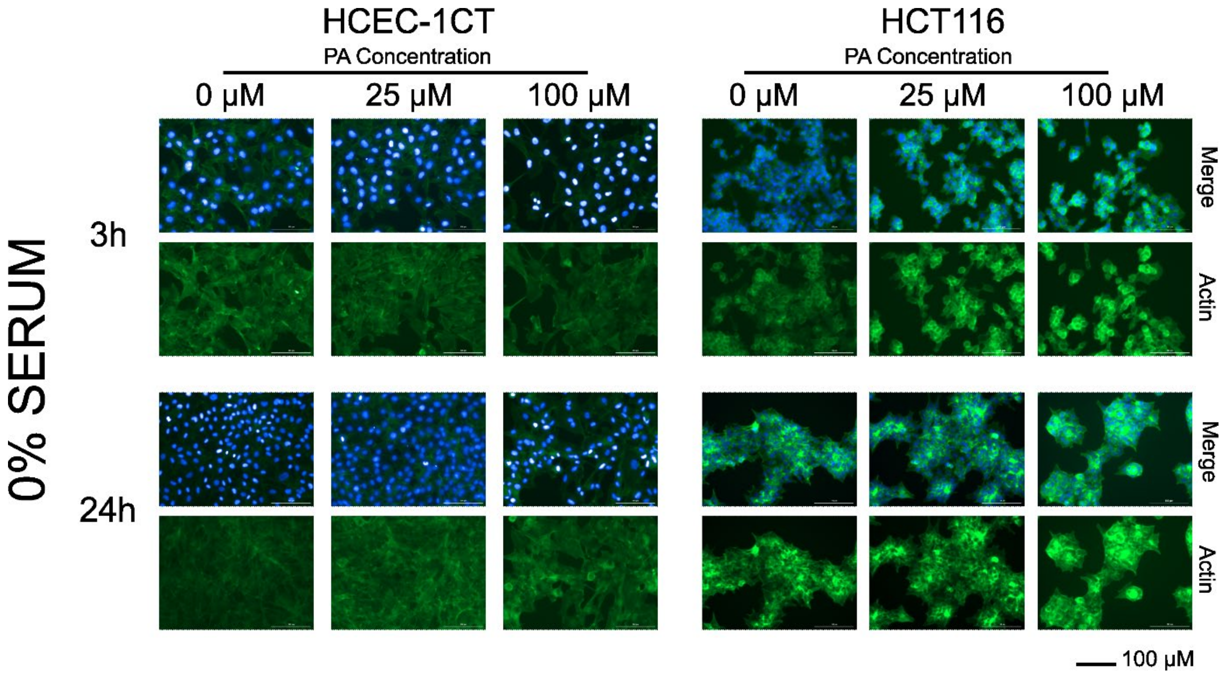

Supplementary Figure 5: Actin appearance after treatment with PA (25 µM, 100 µM) in 0% serum, and application of shear stress (3h, 24h, 2.8 dyn/cm^2) in both HCEC-1CT cells and HCT116 cells. Images were acquired using the Lionheart FX automated microscope (BioTek Instruments Inc., Winooski, VT, USA). Actin cytoskeleton is depicted in green and cell nuclei in blue.

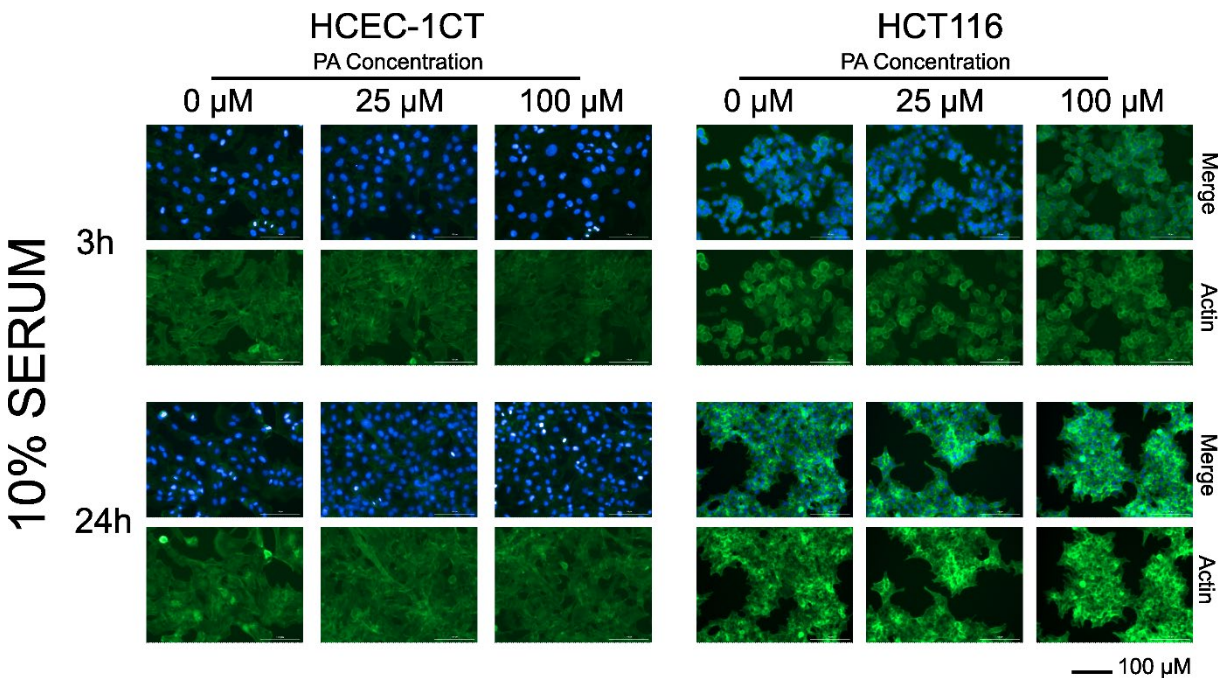

Supplementary Figure 6: Actin intensity after treatment with PA (25 µM, 100 µM) and 10% serum, with the application of shear stress (3h, 24h, 2.8 dyn/cm^2) in both HCEC-1CT cells and HCT116 cells. Images were acquired using the Lionheart FX automated microscope (BioTek Instruments Inc., Winooski, VT, USA). Actin cytoskeleton is depicted in green and cell nuclei in blue.

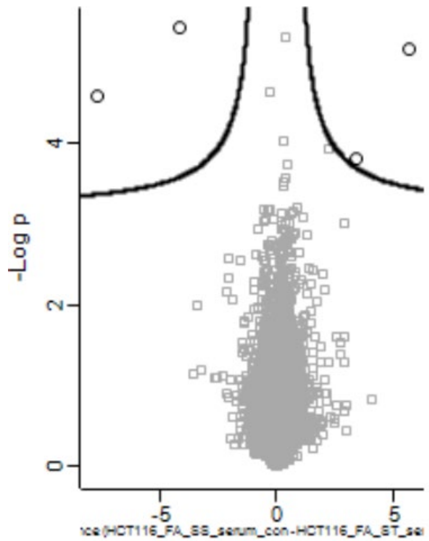

Supplementary Figure 7: Volcano after application of shear stress in presence of 10% serum for HCT116 controls.

Supplementary Table 1: Regulated proteins shown in Figure 4a and 4e and corresponding LFQ intensities.

Gene Name	Protein IDs	24h Shear stress control 0% SERUM			24h Static control 0% SERUM			Difference	- Log (P-value)
		1	2	3	1	2	3		
**DNASE2**	O00115	15,02911472	15,22829247	15,45859909	11,89871597	12,21925926	12,32067299	3,092453	4,19402457
**PDE8A**	O60658	13,79453373	12,81437492	12,53069496	20,83429909	20,65272331	20,59277153	-7,64673	4,40038079
**HMMR**	O75330	18,07583618	18,19361115	18,25402069	13,55601788	12,48744297	12,16616726	5,437947	3,674145
**MARCKS**	P29966	15,65948105	15,13601112	15,64646721	12,94511127	12,62061501	13,46879768	2,469145	2,91814643
**BLM**	P54132	14,72621822	14,51385117	14,63702106	12,99938297	12,36468792	12,79620171	1,905606	3,19445521
**KIF1A**	Q12756	12,05581284	12,64468098	12,03346539	16,3290596	16,63055229	16,84352875	-4,35639	4,19853148
**EZH2**	Q15910	16,67223167	16,62199402	16,17702103	12,39912701	11,80616188	13,10010719	4,055284	3,2494447
**KIF27**	Q86VH2	18,79867363	18,71763039	18,945467	13,96571922	12,59625244	12,26269341	5,879035	3,44019465
**NELFCD**	Q8IXH7-3	15,25192642	15,13318253	14,99276161	12,72063255	12,87811375	11,66074562	2,706126	2,64682889
**ABHD11**	Q8NFV4-5	17,33784103	17,15111923	17,14581108	11,86177635	12,8699131	12,77070045	4,710794	3,87013644
**SYNE2**	Q8WXH0-13	15,57424545	15,33082676	15,33345413	12,66102505	12,99227715	12,38382435	2,7338	3,83648019

**TMX3**	Q96JJ7	12,82095432	13,62696362	13,29853153	17,94572449	17,47831726	17,43204308	-4,36988	3,97187293
**ARAP1**	Q96P48	16,97126198	17,52573395	16,95870209	12,51165104	13,69158649	12,89407253	4,119463	3,32242793
**ATG5**	Q9H1Y0	12,63667679	11,93142796	12,36843681	14,54016495	14,33280563	14,40580654	-2,11408	3,22738002
**SAV1**	Q9H4B6	16,00814438	16,19292831	16,32296753	12,19383049	13,02475929	13,33669376	3,322919	3,14840993
**KIDINS220**	Q9ULH0	16,53232574	16,53762817	16,43879128	13,15440369	12,51109791	12,08310604	3,920046	3,63116848

Supplementary Table 2: Regulated proteins upon application of PA in static conditions and corresponding LFQ intensities.

Gene Name	Protein IDs	24h Static control 0% SERUM			24h Static PA [100 µM] 0% SERUM			Difference	- Log (P-value)
		1	2	3	1	2	3		
**WBSCR16**	Q96I51	12,01328468	12,24966	12,37787	15,91202354	15,83459	15,67441	3,593405406	5,023298152
**HMOX1**	P09601	15,69799042	15,65598	15,40304	17,48005295	17,5317	17,37603	1,876924833	4,275228158

Supplementary Table 3: Regulated proteins shown in Figure 4f and corresponding LFQ intensities.

Gene Name	Protein IDs	24h Shear stress PA [100 µM] 0% SERUM			24h Static PA [100 µM] 0% SERUM			Difference	- Log (P-value)
		1	2	3	1	2	3		
**ZW10**	O43264	15,57047844	15,75465202	15,44201279	16,62152863	16,64091301	16,63248444	-1,04259	3,481732
**RPS4Y1**	P22090	12,17063141	12,28735542	12,09050465	16,51463318	16,99302673	16,08845711	-4,34921	4,077392
**HELZ**	P42694	15,26194763	15,20682335	14,97839737	13,26416016	12,72700119	13,51595116	1,980019	2,872648
**PTEN**	P60484-2	16,45240211	15,87761974	17,13711166	12,84673977	13,22839069	12,43382072	3,652727	2,976336
**ACTB**	P60709	20,77226639	20,85881996	21,02335358	18,48446465	18,30790329	18,29654884	2,521841	4,91254
**KIF1A**	Q12756	13,1328516	12,47592258	11,66872597	17,06770706	16,80069733	16,57478523	-4,38856	3,220409
**MTMR1**	Q13613	18,99291611	18,8629055	18,19418335	13,13154125	13,43365002	12,71812916	5,588895	4,184732
**UBE3C**	Q15386	15,43290043	15,89012909	15,93500423	12,69421101	12,24781132	12,8157196	3,166764	3,750953
**PDLIM3**	Q53GG5	13,69159603	13,78329754	13,78382683	17,23313332	16,47896004	16,06957626	-2,84098	2,939744
**VPS13D**	Q5THJ4	16,7653904	16,84049225	16,89162827	12,52525425	13,24203587	12,81994534	3,970092	4,326347
**LRRC16A**	Q5VZK9-3	12,74322701	12,42572498	13,40003395	15,44111443	14,91833591	15,32052994	-2,37033	2,713387

**C1orf122**	Q6ZSJ8	16,38729286	15,8821888	15,08497429	12,87746906	12,31992817	12,9429121	3,071382	2,701463
**DENND5B**	Q6ZUT9	13,39862633	14,22760391	12,13692093	20,97994423	21,00415421	20,86793327	-7,69629	3,64567
**RNF10**	Q8N5U6	13,02122307	12,53133011	12,72203732	14,86132908	15,06663609	14,61670685	-2,09003	3,384746
**ZNF611**	Q8N823-2	19,04430962	19,40041161	19,12333298	13,70532894	12,71313953	12,54781914	6,200589	4,095803
**SYNE2**	Q8WXH0-13	16,19737053	15,68954277	15,87176323	12,59965611	12,2296772	13,20490456	3,24148	3,268194
**TMX3**	Q96JJ7	13,99728012	15,04473591	13,56620502	17,64455986	17,15541077	17,82494545	-3,3389	2,640708
**ARAP1**	Q96P48	17,16820145	16,41559219	17,06542969	12,44578934	13,25817013	12,40559387	4,17989	3,482749
**TSEN34**	Q9BSV6	13,06410122	13,2204237	12,94860268	15,94699764	15,95517349	16,16195869	-2,94367	5,006577
**SAV1**	Q9H4B6	16,7646122	16,21412849	16,47889519	13,73226261	13,50691128	13,34818554	2,956759	3,965242
**SMARCAL1**	Q9NZC9	13,30538368	12,4688139	12,53639889	15,42304993	15,08659363	14,93902779	-2,37936	2,841284
**KIDINS220**	Q9ULH0	16,22247505	16,55837631	16,54268456	11,93468952	13,34274387	11,83661366	4,06983	2,911131

Supplementary Table 4: Regulated proteins shown in Supplementary Figure 7 and corresponding LFQ intensities.

Gene Name	Protein IDs	24h Shear stress 10% SERUM			24h Static 10% SERUM			Difference	- Log (P-value)
		1	2	3	1	2	3		
**VPS41**	P49754	18,8907516	19,12419128	18,5040936	13,15982914	13,09349251	13,2071352	5,68619651	5,186777758
**DENND5B**	Q6ZUT9	13,48129463	12,52252102	13,16577911	20,35820961	20,84283829	21,06684113	-7,69943141	4,591608132
**U2AF1L4**	Q8WU68	12,423563	12,53048706	12,63618088	16,63026619	16,84352875	16,50896454	-4,13084284	5,438606876
**SAV1**	Q9H4B6	16,03577666	16,30782318	16,03243256	13,10049343	12,64595032	12,31088448	3,439564705	3,816488015
